# Comparative Effects of P2Y12 Inhibitors on Thrombus Biology and Inflammatory Responses in Atherothrombotic Cardiovascular Disease: A Systematic Review of Randomized Controlled Trials

**DOI:** 10.7759/cureus.95888

**Published:** 2025-11-01

**Authors:** Najeeb Ullah, Muhammad Ali, Irum Dad Khan, Mamoon Khan, Hamza Jamil, Nouman Anthony

**Affiliations:** 1 Internal Medicine, Royal Derby Hospital, Derby, GBR; 2 Internal Medicine, Rehman Medical Institute, Peshawar, PAK; 3 Internal Medicine, Northwest General Hospital and Research Centre, Peshawar, PAK; 4 Internal Medicine, Khyber Medical College, Peshawar, PAK; 5 Diabetes and Endocrinology, Royal Derby Hospital, Derby, GBR; 6 Oncology, Northwest General Hospital and Research Centre, Peshawar, PAK

**Keywords:** acute coronary syndrome, cangrelor, clopidogrel, inflammation, p2y12 inhibitors, percutaneous coronary intervention, platelet aggregation, prasugrel, thrombus biology, ticagrelor

## Abstract

This systematic review investigates the biological impact of various P2Y12 receptor inhibitors on thrombus composition and inflammatory activity in patients with acute coronary syndromes undergoing percutaneous coronary intervention (PCI). A comprehensive literature search across four major databases identified four randomized controlled trials that met the inclusion criteria for evidence synthesis. These trials examined ticagrelor, prasugrel, cangrelor, and genotype-guided strategies in comparison to clopidogrel, assessing outcomes such as inflammatory cell infiltration, platelet reactivity, and myocardial reperfusion parameters. Overall, ticagrelor and prasugrel were associated with more favorable modulation of thromboinflammatory and vascular healing markers compared with clopidogrel; these effects were most evident in studies evaluating neutrophil infiltration, myeloperoxidase activity, and early post-PCI ischemic events. However, variations in study design, endpoints, and follow-up duration limited direct comparisons and precluded definitive conclusions. In addition, one mechanistic study protocol describing the assessment of extracellular vesicle-based biomarkers was identified but excluded from the evidence synthesis due to the absence of outcome data. Collectively, the available evidence provides preliminary mechanistic support for the hypothesis that certain P2Y12 inhibitors may exert anti-inflammatory and thrombus-modifying effects beyond their platelet-inhibiting effects. Larger, standardized, and mechanistically focused trials are warranted to validate these findings and guide precision-based antiplatelet therapy in cardiovascular disease.

## Introduction and background

Atherothrombosis is a pathological process characterized by the disruption of atherosclerotic plaques and subsequent thrombus formation, which can lead to acute cardiovascular events such as myocardial infarction and stroke [1]. Platelets play a central role in the development of arterial thrombosis, particularly in response to endothelial injury and plaque rupture. The activation and aggregation of platelets are mediated by various receptors and signaling pathways, among which the P2Y12 receptor holds significant clinical relevance [2].

The P2Y12 receptor is a G protein-coupled receptor expressed on the surface of platelets. Upon stimulation by adenosine diphosphate, it amplifies platelet activation and promotes stable platelet aggregation [3]. Due to its pivotal role in thrombus formation, the P2Y12 receptor has become a major therapeutic target for preventing atherothrombotic events. Several oral antiplatelet agents, including clopidogrel, prasugrel, and ticagrelor, act by inhibiting the P2Y12 receptor, thereby reducing the risk of ischemic complications in patients with acute coronary syndromes (ACS) and those undergoing percutaneous coronary intervention (PCI) [4]. However, the biological effects of P2Y12 inhibition extend beyond platelet aggregation, encompassing the modulation of thrombus composition, inflammatory cell infiltration, and vascular healing, thereby linking platelet biology to inflammation and endothelial repair mechanisms.

While the clinical use of P2Y12 inhibitors is well established, variability in pharmacodynamic response, drug metabolism, and inflammatory modulation has prompted continued research into their comparative effects. This interest spans both ACS and the broader context of atherothrombotic cardiovascular disease, where platelet-driven inflammation contributes to disease progression. The current review specifically evaluates both oral and intravenous P2Y12 inhibitors, including ticagrelor, prasugrel, clopidogrel, and the intravenous, short-acting P2Y12 inhibitor cangrelor, to reflect their mechanistic and translational implications in thrombus biology [5].

In parallel, advancements in precision medicine, particularly genotype-guided antiplatelet strategies, have provided new opportunities to optimize treatment by accounting for CYP2C19 polymorphisms and variable drug responsiveness. In this review, genotype-guided therapy is contextualized from both mechanistic and clinical perspectives, addressing how genetic profiling influences platelet inhibition and thromboinflammatory pathways [6].

Although prior meta-analyses and reviews have compared the clinical efficacy and safety of P2Y12 inhibitors, few have comprehensively synthesized evidence linking these agents to thrombus biology and inflammatory mechanisms. Therefore, a knowledge gap remains in understanding how different P2Y12 inhibitors influence cellular and molecular components of thrombus formation and vascular healing. This systematic review aims to critically evaluate clinical trial evidence on the role of P2Y12 receptor antagonists in atherothrombosis, focusing primarily on their biological and inflammatory effects, while also summarizing key clinical correlations where available.

## Review

Materials and methods

Study Design and Protocol

This systematic review was conducted in accordance with the Preferred Reporting Items for Systematic Reviews and Meta-Analyses (PRISMA 2020) guidelines [7]. The protocol was developed prior to the literature search and outlined the study objectives, eligibility criteria, search strategy, and methods for data extraction and synthesis.

The primary aim of this review was to evaluate randomized controlled trial (RCT) evidence on the biological and inflammatory effects of P2Y12 receptor antagonists, including clopidogrel, ticagrelor, prasugrel, and the intravenous short-acting agent cangrelor, modulating thrombus composition, inflammatory markers, and related mechanistic parameters in patients with ACS, particularly those undergoing PCI. Rather than focusing on clinical outcome endpoints such as mortality or reinfarction, this review emphasizes mechanistic and translational aspects of P2Y12 inhibition, including platelet activation, inflammatory cell infiltration, and extracellular vesicle (EV) dynamics. This scope aligns with the nature of the included studies, which predominantly explored pathophysiological and biomarker-based outcomes.

The review protocol was not prospectively registered in the International Prospective Register of Systematic Reviews (PROSPERO). This decision was made because the review was narrowly mechanistic in scope and did not involve patient-level meta-analysis or public health outcomes, which are primary priorities for PROSPERO registration. However, all steps of the review, including eligibility determination, data extraction, and bias assessment, were predefined and conducted in accordance with PRISMA standards to ensure methodological transparency and reproducibility.

Eligibility Criteria (PICO Framework)

The inclusion criteria were established based on the PICO (population, intervention, comparator, and outcome) framework [8]. The population included adult patients diagnosed with atherothrombosis or ACS, such as ST-elevation myocardial infarction (STEMI), non-STEMI, or unstable angina. The intervention involved administration of P2Y12 receptor inhibitors (ticagrelor, prasugrel, clopidogrel, or cangrelor), either as part of dual antiplatelet therapy or monotherapy. Comparators included other P2Y12 inhibitors or standard therapy with clopidogrel. The outcomes of interest were primarily biological and mechanistic parameters, such as inflammatory cell infiltration within the thrombus, platelet activation, EV concentrations, and myocardial reperfusion indices (e.g., TIMI flow, myocardial blush grade). Although ischemic and bleeding events were recorded when reported, these were analyzed descriptively rather than as primary endpoints. Only RCTs published in peer-reviewed journals and written in English were included.

During the full-text screening process, a small number of potentially relevant studies were excluded due to language (non-English) or unpublished/preprint status. These exclusions were made to maintain methodological consistency, ensure data verifiability, and focus on peer-reviewed evidence. However, the authors acknowledge that this language restriction may have led to the omission of some region-specific or non-English studies, which could introduce minor publication bias and limit the comprehensiveness of the evidence base. This consideration has been reflected in the limitations section to enhance transparency and critical appraisal of the review’s findings.

Search Strategy and Data Sources

A comprehensive literature search was conducted across multiple databases, including PubMed, Embase, Scopus, and the Cochrane Central Register of Controlled Trials (CENTRAL), encompassing publications from the database's inception to July 2025. The search strategy combined Medical Subject Headings (MeSH) and free-text keywords such as “P2Y12 receptor,” “clopidogrel,” “ticagrelor,” “prasugrel,” “cangrelor,” “atherothrombosis,” “platelet aggregation,” “PCI,” and “inflammation.” The reference lists of included studies were also manually screened to identify additional eligible articles.

Study Selection and Data Extraction

Two reviewers independently screened titles and abstracts for eligibility. Full-text articles of potentially relevant studies were then assessed using predefined inclusion and exclusion criteria. Discrepancies between reviewers were resolved by discussion or consultation with a third reviewer. A standardized data extraction form was used to collect relevant information from each included study, including author, publication year, study design, sample size, population characteristics, interventions, comparators, outcomes measured, and follow-up duration.

Quality Assessment

The methodological quality and risk of bias of the included RCTs were assessed using the Cochrane Risk of Bias 2.0 tool (RoB 2.0; Cochrane, London, UK) [9]. Each study was evaluated across five domains: randomization process, deviations from intended interventions, missing outcome data, measurement of outcomes, and selection of the reported results. Each domain was rated as “low risk,” “some concerns,” or “high risk,” and an overall risk of bias was assigned accordingly.

Given that only peer-reviewed trials were included, a formal publication bias assessment (e.g., funnel plot analysis) was not performed, as the limited number of eligible studies (n = 4) precluded a meaningful evaluation. However, potential publication bias was minimized through comprehensive database searches and manual screening of references. Across the included trials, the most frequent sources of bias were selective reporting and incomplete information on randomization procedures, typically classified as “some concerns.” None of the studies were rated as “high risk” overall, indicating generally strong methodological quality. However, one protocol-only study exhibited multiple domains with partial data and thus warranted cautious interpretation.

Data Synthesis

Due to heterogeneity in outcome measures, drug regimens, and reporting styles, a qualitative synthesis was performed. Studies were grouped thematically based on their primary focus, such as thrombus inflammation, reperfusion, pharmacogenomics, or extracellular biomarkers, and analyzed narratively to compare efficacy and mechanistic insights of different P2Y12 inhibitors. A quantitative meta-analysis was not conducted due to the diversity of outcome definitions and the limited availability of comparable data across trials.

Results

Study Selection Process

**Figure 1:**
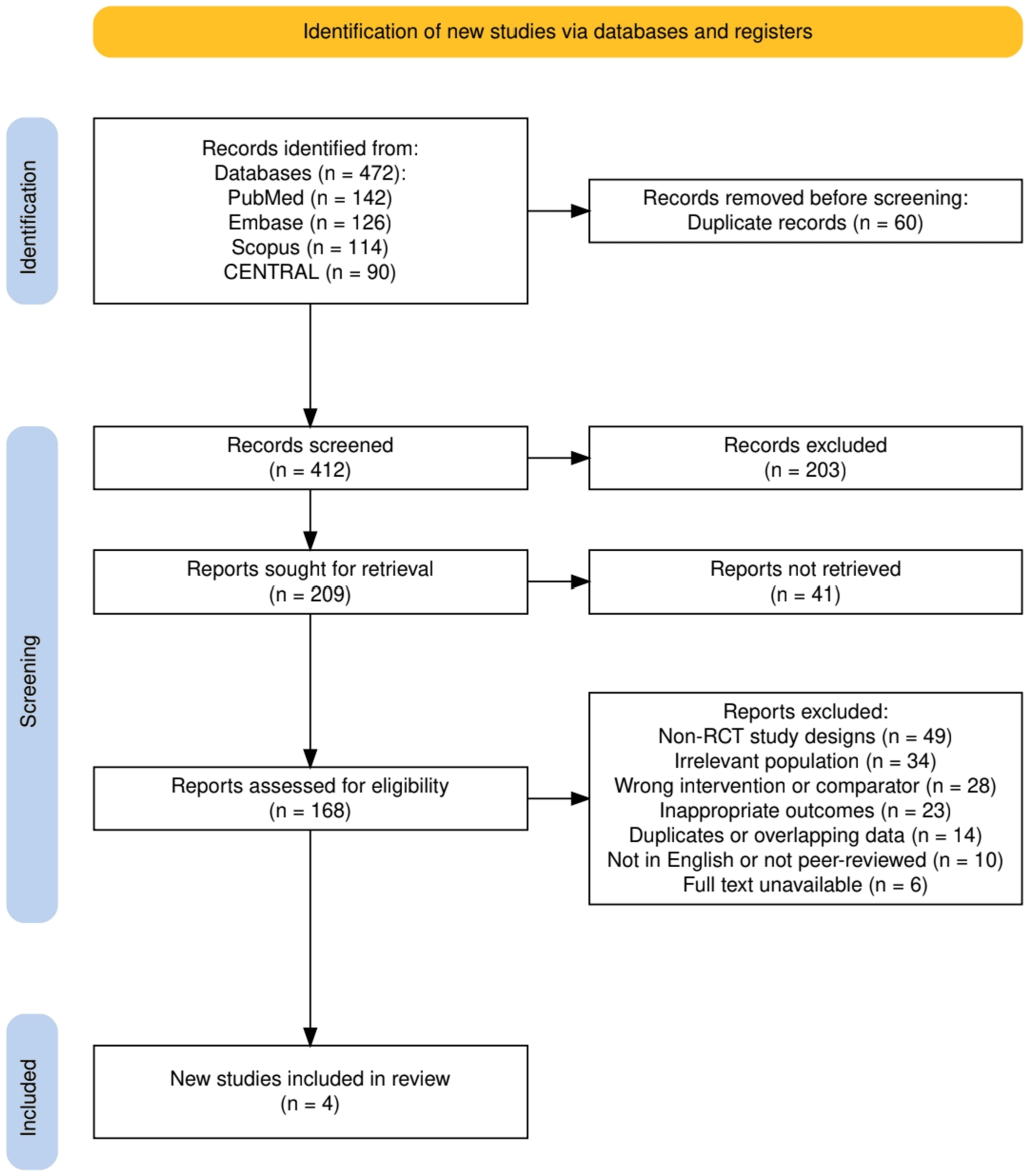
PRISMA flowchart representing the study selection process PRISMA: Preferred Reporting Items for Systematic Reviews and Meta-Analyses

The selection process adhered to the PRISMA 2020 guidelines and is illustrated in Figure 1. A total of 472 records were identified through database searches, including PubMed (n = 142), Embase (n = 126), Scopus (n = 114), and CENTRAL (n = 90). After removing 60 duplicates, 412 records were screened based on titles and abstracts, resulting in the exclusion of 203 articles. The full texts of 209 reports were sought, but 41 could not be retrieved due to restricted access (paywalled or unavailable institutional subscriptions), incomplete publication formats (conference abstracts or early online notices without full data), or duplication across registries. Among the 168 reports assessed for eligibility, 164 were excluded for reasons including non-RCT study designs (n = 49), irrelevant population (n = 34), inappropriate interventions or comparators (n = 28), outcomes not aligned with inclusion criteria (n = 23), duplicate or overlapping data (n = 14), non-English or non-peer-reviewed articles (n = 10), and unavailability of full texts (n = 7). Ultimately, four studies were included in the final qualitative synthesis, and one protocol-only publication was described narratively but excluded from evidence synthesis due to the absence of outcome data. Although these non-retrievable reports represented a modest portion of the initially screened literature, their exclusion may introduce retrieval bias. However, most were preliminary or duplicate records without complete trial data, and their omission is unlikely to have materially affected the qualitative synthesis.

Characteristics of the Selected Studies

Table 1 presents an overview of the four RCTs included in this systematic review, highlighting key methodological and clinical attributes. All studies focused on adult patients diagnosed with ACS, particularly those undergoing PCI. Sample sizes varied significantly, ranging from 47 to over 11,000 participants, reflecting both pilot investigations and large-scale multicenter trials. Various P2Y12 inhibitors were evaluated, including ticagrelor, prasugrel, and cangrelor, as well as genotype-guided therapy, with clopidogrel serving as the most common comparator. Primary outcomes encompassed a diverse set of mechanistic and clinical endpoints, including inflammatory cell infiltration in thrombus material, thrombus burden assessed via optical coherence tomography (OCT), EV concentrations, and early ischemic or platelet reactivity events. Follow-up durations were generally short, often limited to the in-hospital or early post-procedural period, aligning with the acute nature of the interventions and endpoints measured. This heterogeneity underscores the diverse range of approaches employed to evaluate the role of P2Y12 receptor inhibitors in modulating thrombo-inflammatory pathways and clinical outcomes within the context of atherothrombosis.

**Table 1:** Characteristics of the included studies in the review STEMI: ST-elevation myocardial infarction, PCI: percutaneous coronary intervention, MPO: myeloperoxidase, OCT: optical coherence tomography, MBG: myocardial blush grade, TIMI: thrombolysis in myocardial infarction, IV: intravenous, MI: myocardial infarction, EVs: extracellular vesicles, AMI: acute myocardial infarction, ACS: acute coronary syndrome, GG: genotype-guided group, SG: standard (control) group, HTPR: high on-treatment platelet reactivity, RCT: randomized controlled trial

First author (year)	Study design	Sample size	Population characteristics	P2Y12 inhibitor(s)	Comparator	Primary outcome(s)	Follow-up duration
Shen et al. (2022) [10]	RCT	47 (completed)	STEMI patients undergoing PCI with thrombus aspiration	Ticagrelor (180 mg loading dose)	Clopidogrel (600 mg loading dose)	Inflammatory cell infiltration in thrombus (total inflammatory cells, neutrophils, MPO-positive cells, monocytes)	Not explicitly stated (single in-hospital evaluation)
Di Vito et al. (2016) [11]	RCT	128 (clopidogrel: 44, prasugrel: 45, ticagrelor: 39)	STEMI patients undergoing PCI (COCTAIL II trial)	Prasugrel, ticagrelor	Clopidogrel	Residual thrombus burden (OCT-based), reperfusion indexes (MBG, TIMI flow, ST-segment resolution)	Not explicitly stated (post-procedure evaluation)
Cavender et al. (2022) [12]	RCT	11,145	Patients undergoing PCI (CHAMPION PHOENIX trial)	Cangrelor (IV)	Clopidogrel	Composite ischemic events within 2 hours: death, MI, revascularization, stent thrombosis	Up to 48 hours (primary analysis focused on the first 2 hours)
Tam et al. (2017) [13]	RCT	132 ACS patients (GG: 65, SG: 67)	Chinese patients with ACS	Genotype-guided ticagrelor or clopidogrel	Standard care with clopidogrel	High on-HTPR at 24 hours and 1 month	1 month

Quality Assessment

The risk of bias assessment was conducted using the RoB 2.0 [9]. Of the four included trials, two were rated as having a low risk of bias, demonstrating strong methodological quality. At the same time, the remaining two showed some concerns, primarily related to randomization procedures and selective reporting. Overall, the evidence was considered to be of low to moderate risk of bias, with conclusions primarily guided by studies of higher quality. The RoB 2.0 tool is openly available for academic use and was applied without the need for special licensing.

**Table 2:** Risk of bias assessment of the included studies in the review The risk of bias assessment was conducted using the Cochrane Risk of Bias 2.0 tool [9].

Study (first author, year)	Randomization process	Deviations from intended interventions	Missing outcome data	Measurement of the outcome	Selection of reported result	Overall risk of bias
Shen et al. (2022) [10]	Low risk	Low risk	Low risk	Low risk	Low risk	Low risk
Di Vito et al. (2016) [11]	Some concerns	Low risk	Low risk	Low risk	Some concerns	Some concerns
Cavender et al. (2022) [12]	Low risk	Low risk	Low risk	Low risk	Low risk	Low risk
Tam et al. (2017) [13]	Low risk	Low risk	Low risk	Low risk	Some concerns	Low risk

Discussion

Mechanistic and Clinical Implications of P2Y12 Inhibitors

This systematic review consolidates recent clinical trial evidence assessing the impact of P2Y12 receptor antagonists on not only platelet aggregation but also the biological composition of thrombi, reperfusion parameters, and individualized pharmacogenomic responses. Preliminary mechanistic evidence suggests that more potent agents such as ticagrelor and prasugrel may exert anti-inflammatory effects beyond platelet inhibition, although definitive clinical correlations remain limited. For instance, Shen et al. [10] reported in a small single-center RCT (n = 47) that ticagrelor significantly reduced infiltration of neutrophils and MPO-positive cells in thrombi aspirated from STEMI patients, indicating a potential modulatory role in thromboinflammation. However, these findings are based on mechanistic markers rather than clinical endpoints, and larger multicenter trials are needed to validate whether such biological effects translate into measurable benefits in outcomes.

In contrast, the CHAMPION PHOENIX trial (Cavender et al. [12]), a large RCT with over 11,000 participants, demonstrated that cangrelor, an intravenous short-acting P2Y12 inhibitor, significantly reduced early ischemic events within two hours of PCI. While this supports the clinical utility of rapid platelet inhibition in acute settings, it primarily reflects pharmacodynamic potency rather than direct anti-inflammatory action. Similarly, studies such as Gasecka et al. [14] introduced EV concentrations as potential mechanistic biomarkers of platelet activation and inflammation following AMI. However, their prognostic value remains investigational and has yet to be linked to clinical outcomes.

Taken together, the anti-inflammatory effects observed across P2Y12 inhibitors appear to be drug-specific rather than class-specific, with ticagrelor showing the most consistent mechanistic signals. Nonetheless, current evidence arises from small, short-term studies with limited power to detect downstream clinical improvements. Thus, while mechanistic data indicate potential roles for P2Y12 inhibitors in modulating vascular inflammation and healing, these hypotheses require confirmation through larger, mechanistically integrated clinical trials designed to correlate biological modulation with tangible outcome benefits.

Pharmacologic Distinctions and Therapeutic Optimization

Comparative data suggest that not all P2Y12 inhibitors are pharmacologically equal, and their differences carry critical clinical implications. Ticagrelor, for instance, acts directly without requiring hepatic activation, unlike clopidogrel, which is a prodrug subject to interindividual metabolic variability, a factor especially relevant in East Asian populations, where CYP2C19 polymorphisms are prevalent [15,16]. Tam et al. [13] demonstrated that a rapid genotype-guided strategy significantly reduced high on-treatment platelet reactivity, supporting the shift toward personalized antiplatelet regimens. Additionally, the reversible inhibition offered by ticagrelor and cangrelor may provide safer options for patients requiring urgent surgery, as platelet function recovers quickly upon drug cessation [17]. In contrast, irreversible agents such as clopidogrel and prasugrel may increase perioperative bleeding risk. These distinctions underline the need for individualized agent selection based not only on ischemic risk but also on procedural timing, bleeding risk, and pharmacogenetic profiles.

Emerging Concepts in Platelet Biology and Personalized Medicine

Antiplatelet agents and inflammation: Emerging evidence suggests that P2Y12 inhibitors, particularly ticagrelor, may influence not only thrombosis but also inflammatory pathways, a phenomenon increasingly described as thromboinflammation [18]. This dual action could be pivotal in mitigating downstream myocardial injury and systemic complications following STEMI. Shen et al. [10] demonstrated a significant reduction in inflammatory cell infiltration within thrombi with ticagrelor compared to clopidogrel. To further understand this interaction, future clinical trials should incorporate inflammatory biomarkers, such as high-sensitivity C-reactive protein and interleukin-6 (IL-6), as secondary endpoints, potentially allowing for risk stratification and therapy tailoring based on the patient’s inflammatory profile.

The AFFECT EV study protocol, as introduced by Gasecka et al., utilizes EVs as novel biomarkers of platelet activation and inflammation [14]. EVs released from activated platelets, leukocytes, and endothelial cells may serve as real-time indicators of thrombotic activity and antiplatelet efficacy. Measuring EV concentrations in response to P2Y12 inhibition, particularly with ticagrelor versus clopidogrel, may provide an innovative approach to monitoring therapeutic response and residual platelet reactivity. However, the reliability and clinical utility of EV measurements remain limited, as current detection techniques such as flow cytometry and nanoparticle tracking analysis vary in sensitivity, standardization, and reproducibility. At present, EV assessment is considered an exploratory mechanistic tool rather than a validated clinical parameter. Future well-designed trials using standardized methodologies and correlating EV dynamics with clinical outcomes are needed to determine their prognostic value and potential role in guiding individualized antiplatelet therapy [19].

Pharmacogenomics in ACS: The clinical importance of pharmacogenomic profiling in ACS management is gaining recognition, particularly in populations with high prevalence of CYP2C19 loss-of-function alleles, such as East Asians. Tam et al. [13] demonstrated that a genotype-guided approach significantly reduced high on-treatment platelet reactivity compared to standard clopidogrel therapy. Despite these benefits, genotype-guided selection remains underutilized. Its integration into future ACS guidelines could revolutionize antiplatelet therapy by minimizing adverse events and enhancing treatment precision, especially in patients undergoing PCI, where immediate efficacy is critical [20].

Timing of thrombosis events: Insights from the CHAMPION PHOENIX trial (Cavender et al., [12]) underscore that the majority of ischemic events post-PCI occur within the first two hours, a critical window that demands rapid platelet inhibition. Cangrelor, with its immediate onset and reversibility, was shown to significantly reduce these early events compared to clopidogrel. This temporal clustering of thrombotic risk supports the strategic use of intraprocedural P2Y12 inhibitors, particularly in high-risk PCI cases or in patients who are P2Y12-inhibitor naïve [21]. Future protocols may benefit from integrating pharmacokinetic and pharmacodynamic timing models into decisions regarding antiplatelet regimens.

Clinical Implications

The findings of this systematic review prompt a critical re-evaluation of clopidogrel as the default first-line P2Y12 inhibitor, particularly in patients with STEMI and those with high thrombus burden. The variability in clopidogrel metabolism, largely influenced by CYP2C19 genetic polymorphisms, contributes to inconsistent platelet inhibition and higher rates of adverse ischemic events [22]. In contrast, newer agents such as ticagrelor, prasugrel, and cangrelor demonstrate more predictable pharmacodynamic profiles and faster onset of action. However, their clinical application requires nuanced judgment, as potency must be weighed against the risk of bleeding, procedural urgency, and patient-specific factors such as comorbidities and surgical needs.

Beyond their antiplatelet activity, this review highlights that P2Y12 inhibitors may exert biological effects on thrombus composition, inflammatory infiltration, and vascular healing. Evidence from mechanistic trials suggests that these agents can influence thromboinflammatory pathways, potentially reducing neutrophil activity, oxidative stress, and the release of EVs within atherothrombotic lesions. This expanded understanding of P2Y12 inhibition underscores the need to view antiplatelet therapy not merely as a means of preventing aggregation but as a modulatory tool capable of influencing vascular biology and post-PCI myocardial recovery. Integrating such mechanistic insights into clinical decision-making could redefine how antiplatelet therapy is optimized for different stages of coronary disease and patient phenotypes [23].

A key clinical implication emerging from this synthesis is the necessity for individualized therapy. The traditional “one-size-fits-all” model of dual antiplatelet therapy may not be suitable for all patients with ACS. Factors such as thrombus morphology, platelet turnover, genetic predisposition, and systemic inflammatory state should inform the selection of the most appropriate agent. Incorporating pharmacogenomic data and point-of-care platelet function testing could enable clinicians to personalize treatment regimens, choosing, for instance, ticagrelor for rapid, reversible inhibition in surgical candidates or prasugrel for high-thrombus-burden STEMI cases. The transition toward personalized cardiovascular care, guided by biologic and genomic profiling, represents a major step forward in optimizing efficacy while minimizing bleeding risk.

Limitations of the Evidence

While the included trials provide valuable mechanistic insights, several limitations warrant consideration. Most studies, including those by Shen et al. [10] and Cavender et al. [12], had short follow-up durations and small sample sizes, which limited the ability to draw long-term and generalizable conclusions. The heterogeneity of endpoints and the limited number of RCTs mean that mechanistic hypotheses, including those related to EVs, pharmacogenomics, and inflammatory modulation, remain exploratory rather than confirmatory. Publication bias cannot be excluded, given the reliance on peer-reviewed, English-language articles and the fact that the included populations were predominantly of European and East Asian origin, which limits ethnic diversity. The AFFECT EV trial by Gasecka et al. [14], available only as a study protocol, was described qualitatively but excluded from data synthesis to avoid inflating the certainty of the results. Practical trade-offs, such as higher bleeding risk, cost, and limited accessibility of newer P2Y12 inhibitors like ticagrelor, prasugrel, and cangrelor [10,12,14,19], must also be acknowledged, as these factors influence the real-world applicability. Collectively, these limitations highlight the need for larger, more diverse, and outcome-focused studies to confirm the mechanistic findings presented in this review.

Future Directions and Research Priorities

Future research should aim to clarify the mechanistic link between platelet inhibition, thrombus inflammation, and clinical recovery through well-designed, multicenter RCTs that integrate standardized biological endpoints, including EV concentrations, neutrophil activation, and cytokine profiles [14,19]. Establishing uniform definitions and quantification methods for thrombo-inflammatory markers would facilitate cross-trial comparisons and enable future meta-analyses. The use of advanced imaging modalities, such as OCT and intravascular ultrasound, can further elucidate the dynamics of thrombus remodeling and endothelial healing in response to P2Y12 inhibition [10,12]. Moreover, implementing real-time pharmacogenomic profiling as a point-of-care tool to guide rapid therapy selection in acute PCI settings remains an important yet resource-dependent goal, necessitating an evaluation of its cost-effectiveness and feasibility across diverse healthcare systems. Collaborative translational frameworks that unite molecular research with clinical cardiology, through shared biorepositories, standardized imaging protocols, and multicenter networks, will be crucial for bridging laboratory findings with clinical outcomes and advancing precision antiplatelet therapy in atherothrombotic disease.

## Conclusions

This systematic review provides hypothesis-generating evidence suggesting that P2Y12 receptor antagonists may influence not only platelet inhibition but also thrombus biology and inflammatory pathways in ACS. Among the mechanistic insights reviewed, modulation of neutrophil infiltration and EV dynamics currently holds the strongest potential clinical relevance. However, findings remain preliminary and require validation in larger, outcome-focused trials. Given the limited number and heterogeneity of included studies, the overall certainty of evidence is moderate, and conclusions should be interpreted with caution. Future research should aim to operationalize these mechanistic observations through the use of standardized biomarkers, pharmacogenomic-guided therapy, and imaging-based monitoring, thereby facilitating their integration into clinical treatment algorithms. Substantial knowledge gaps persist regarding the long-term effects, cost-effectiveness, and real-world feasibility of these strategies, underscoring the need for continued translational and multicenter investigations before they can be routinely adopted in cardiovascular care.
